# “This is not a doctors thing, it is witchcraft” - A case report of acute psychosis concomitant to primary hyperparathyroidism

**DOI:** 10.1192/j.eurpsy.2021.672

**Published:** 2021-08-13

**Authors:** L. Lopes, B. Moura, S. Pereira

**Affiliations:** Department Of Psychiatry And Mental Health, Centro Hospitalar de Vila Nova de Gaia e Espinho, Vila Nova de Gaia, Portugal

**Keywords:** hypercalcemia, hyperparathyroidism, old age, psychosis

## Abstract

**Introduction:**

Primary hyperparathyroidism (PHPT), usually caused by a parathyroid adenoma, is characterized by a pathologically high secretion of parathyroid hormone and consequent hypercalcemia. PHPT has a high prevalence among elderly patients and might be responsible for neuropsychiatric symptoms.

**Objectives:**

We aim to report the rare manifestation of acute psychosis accompanying a PHPT diagnosis, and to discuss the neurobiological relationship between hyperparathyroidism, hypercalcaemia and neuropsychiatric symptoms.

**Methods:**

We present a clinical case based on patient’s history and clinical data, along with a literature review on PHPT neuropsychiatric symptons.

**Results:**

We present the case of a 68-year-old man diagnosed with PHPT in November 2019. He was brought up to psychiatric evaluation for the first time in May 2020 upon behavioral changes (aggressiveness and bizarre rituals). The patient described the sensation of burns scattered throughout the body since January 2020, felling anxious and frightened, sleeping poorly and progressive social isolation. He presented delusional ideas of mystical and paranoid content. No significant cognitive impairments were found. The patient’s psychosis was partially responsive to atypical antipsychotics. He’s waiting for surgery. Hypercalcaemia might manifest as mood disorders, cognitive changes and rarely as acute psychosis. Although there is not yet a clear mechanism to explain it, high calcium levels seem to cause neurotoxicity and neurotransmission dysfunction. Restoration of normal calcium levels tend to resolve neuropsychiatric symptoms, but in PHPT parathyroidectomy is usually recommended.

**Conclusions:**

Neuropsychiatric symptoms are responsible for great disability, and demand an organic in-depth investigation. A multidisciplinary team approach must always be considered in the management of such conditions.

